# Establishing the relationship between non-human primates and mangrove forests at the global, national, and local scales

**DOI:** 10.1371/journal.pone.0277440

**Published:** 2022-11-11

**Authors:** Stuart E. Hamilton, Andrea Presotto, Arthur J. Lembo

**Affiliations:** 1 Department of Coastal Studies, East Carolina University, Greenville, NC, United States of America; 2 Biological Sciences Graduate Program, Federal University of Maranhão, Chapadinha, Maranhão, Brazil; 3 Geographic Cognition and Conservation Lab, Salisbury University, Salisbury, MD, United States of America; 4 Eastern Shore Regional GIS Cooperative, Salisbury University, Salisbury, MD, United States of America; Sichuan University, CHINA

## Abstract

Global and spatially explicit information about the interaction between habitat and wildlife species is critical to enhancing conservation efforts. Despite the recognized importance of mangrove forests to non-human primates, the relationship between the two lacks understanding. To counter this, we created the MangPrim-21 database to map and measure the locations of interactions between all non-human primates and all mangrove forests globally. We report our findings across the global, national, and local scales for all inventoried non-human primates and all inventoried mangrove forests. Globally, we find that half of all non-primates potentially use mangrove forests, and more than half of the global mangrove forest falls within the delineated range of at least one non-human primate species. Nationally, we find that Indonesia, Madagascar, Brazil, Cameroon, and Malaysia likely have the most non-human primate and mangrove forest interactions. At the subnational level, we find that several discrete locations in Kalimantan are critical to both mangrove forests and non-human primates. The MangPrim-21 database provides a globally consistent and locally applicable database of non-human primate and mangrove forest interactions. The results presented have broader implications for non-human primate and mangrove conservation and global actions to protect both. Additionally, our results raise questions about the idea that non-human primates primarily use mangrove forests as a refuge from human encroachment and habitat degradation.

## Introduction

The broad idea that non-human primate species (NHPS) increase their use of mangrove forests only when their traditional terrestrial habitats are threatened is a dominant concept in recent primate literature [1–5]. In such studies, mangrove forests are viewed primarily as a refuge for NHPS due to increased human activities such as deforestation, agricultural expansion, and general encroachment by urban development that threaten their primary habitat [6]. Despite such assertations, the role of mangrove forests as NHPS refuge primarily driven by terrestrial forest degradation is not well documented. The idea of mangrove forests acting as NHPS refuge ordinally appears to be based on a collation of case studies, dominated by studies of various *Colobus* species in Africa [1, 7, 8] and some non-*Colobus* species in Africa [1, 9, 10]. This refuge viewpoint has expanded to other NHPS in the neotropics [11, 12] and Asia [1, 13].

Mangrove Forests and NHPS share similar global geographic extents (S1A and S1B Figs in S1 Appendix) [14–16], and they appeared at about the same time in the geologic record. Mangrove forests developed in the late Cretaceous, approximately 65 Mya, with the most common genus *Rhizophora* appearing 47.8–54.6 Mya [17]. The earliest agreed-upon primate in the fossil record date from 50–55 Mya [18, 19], and several primate fossils indicate that they were present in floodplains and coastal zones [20, 21] where mangroves reside. Indeed, arguably the oldest primate fossil observed, *Altiatlasius koulchii*, was discovered in 60 Mya old coastal sediments in present-day Africa [20]. Therefore, primate associations with coastal environments were likely present from the outset [22].

Much of the literature that examines mangrove forests and NHPS has historically existed in discrete silos across differing disciplines. For example, when primatologists observe NHPS, nearby mangrove forests are often noted, but the use of mangrove forests as NHPS habitat is not the primary focus of studies [23]. Conversely, when mangrove forests are studied, the use of mangrove forests as critical NHPS habitat is rarely the focus of the study, yet NHPS are often noted as present. Additionally, there has existed a historic fallacy in the NHPS and forest conservation literature stating that mangrove forests are less productive, less impacted, and less threatened than terrestrial forests [1, 24–26] and therefore, more likely to act as a refuge rather than a primary habitat to NHPS [1–5]. However, this view is misplaced and outdated, as mangrove forests are among the most productive ecosystems globally [27–29], and primate studies have been slow to recognize this fact.

Additionally, mangroves are as heavily threatened and impacted by anthropogenic forces as terrestrial forests, which questions the principle that mangroves act primarily as a refuge, not primary habitat to NHPS. For example, from the 1970s to the 2000s, across eight of the most extensive mangrove forests holding nations, mangrove forest loss was estimated at 52 percent [30, 31]. Indeed, humans have exploited mangrove forests and deforested them from prehistory to the present [18]. This historic misconception of the lack of productivity of mangrove forests and the belief that mangrove forests are less impacted by human activity than terrestrial forests may have led to the importance of mangrove forests as NHPS primary habitat being understated and the mangrove forests’ role as a refuge to NHPS when terrestrial habitats are threatened NHPS being overstated.

Numerous partialities occur when examining the role of NHPS in interacting with mangrove forests. Firstly, few case studies demonstrate the NHPS migration from threatened terrestrial environments to mangrove forests over suitable decadal timeframes that coincide with the period of human encroachment or habitat loss. Indeed, we could find none. Primate-mangrove studies are generally limited spatiotemporally, focusing on NHPS groups or individual species at a single study site [32] over a short duration, often just a single spatiotemporal snapshot. Additionally, past and current observations of NHPS are often biased against observation within mangrove forests due to the difficulty of traversing and working in the flooded terrain [1, 33]. When NHPS are known to inhabit mangrove forests, observations are often taken outside the mangrove forests for this reason [34, 35].

Since about 2010, there has been an increase in the inventorying of NHPS use of mangrove forests [e.g. 1, 4, 36–38]. The lemurs of Madagascar provide an example of this development. Only one lemur was reported to utilize the mangrove forests in the 1950s; this increased to two lemur species by 1994, and by 2016, 23 lemur species were known to use mangrove forests [3, 39]. Indeed, recent NHPS literature demonstrates the relationship between NHPS and mangrove forests for the Neotropics [11], Africa [1, 38], and Asia [1, 40], in addition to an inventory of the use of mangroves by the lemurs of Madagascar [3] and other regional collections [4, 39, 41]. Some of these relationships reinforce the local knowledge that NHPS inhabit mangrove forests. For instance, *Macaca fascicularis* is known locally in Thailand as the mangrove monkey. It is known that *Nasalis larvatus* has its primary habitat within mangrove forests. Other NHPS mangrove forest relationships are noted, such as the critically endangered *Piliocolobus kirkii* [42, 43] and *Piliocolobus epieni* [44, 45], as well as two capuchin species, the tool-using capuchin species, *Sapajus libidinosus* [11, 46] and *S*. *apella* [47]. In contrast, the global status of the relationship between all NHPS and mangrove forests remains unknown or has limited documentation.

This paper provides the first global reporting of interactions between NHPS and mangrove forests. At high spatial resolutions, we account for all known NHPS and all mangrove forests globally. We synthesize the 30 m global remotely sensed mangrove change database known as CGMFC-21 [15] and the entire range data for all 511 NHPS from the IUCN [14]. We use parallelized computing resources to establish the potential interactions between all NHPS and all mangrove forests at the global, national, and local scales. We then verify these predicted interactions with a meta-analysis of the NHPS literature. The resulting MangPrim-21 database provides spatially explicit information on the interactions between mangrove forests and NHPS, including accounting for the presence of endangered NHPS in mangrove forests. MangPrim-21 is freely available to researchers for further analysis and replication.

## Materials and methods

MangPrim-21 is a data synthesis between the global mangrove database CGMFC-21 [15] and the ranges of all 511 NHPS as defined by the IUCN [14]. CGMFC-21 is derived from remotely sensed data, has a resolution of 1 arc-second or approximately 30 m at the equator, and is stored in raster format. CGMFC-21 is recognized as the only global high-resolution annual mangrove change database that exists [18]. To increase the temporal overlap between the two datasets, CGMFC-21 was extended from its published version end date of 2012 to 2014. Species experts spatially delineate the IUCN NHPS ranges, and the dataset comprises hundreds of scale-independent polygons. The average year of IUCN NHPS delineation is 2014.

The CGMFC-21 and IUCN primates range datasets were supplemented by a meta-analysis of the academic literature that extracted the observed NHPS use of mangrove forests as habitat. The mangrove database has over 126 million unique locations where the mangrove area is observed across 137,760 km^2^, covering much of the world’s tropical coastlines. The NHPS polygonal dataset is constructed using almost 3.4 million spatially explicit vertices, and the combined spatial extent is 45,708,585 km^2^ covering much of the terrestrial tropics. Due to the geographic extent and number of observations across these datasets, unique spatial SQL parallel processing solutions, often referred to as big-data analytics, were used to synthesize the databases. Other global ancillary datasets were used, including the World Database on Protected Areas [48].

The literature search was conducted using Google Scholar, web searches, ISI, and citations in published papers. The search is best considered a literature search available to a US academic. We searched the literature for all the NHPS that our data indicated overlapped mangrove forests. We used the species’ scientific name plus the word mangrove, as "*Sapajus libidinosus* AND mangroves." We also used the species’ popular name in mangroves, such as "bearded capuchins." If the resulting literature did not clearly state the species’ presence in mangroves, we combined the scientific and popular name with the following terms: riverine forest, coastal zones, coastal forests, and swamps. If scientific papers were not found, we included the grey literature based only on the species’ scientific and popular name using Google.

Additionally, we searched a combination of the primate scientific name plus mangroves in Portuguese for neotropical primates (mangue or florestas de manguezal), scientific name plus mangroves for species living in Indonesia (bakau is mangrove in Indonesian), and Spanish (mangle). We used the species name, scientific and popular, when the results were negative. We indexed the publication in order to check if the species’ geographic distribution showed any other term that could suggest the presence of mangrove forests. Additionally, we found publications in French. When the scientific name appeared in non-author languages, the author languages are English, Spanish, and Portuguese, we used Google Translate to translate the publication into English. This method allowed us to access sources in diverse countries. We also checked all maps available in each paper or publication to understand the species distribution according to the maps.

Once appropriate data checks and coordinate matching were complete, the initial synthesis involved iterating through each of the 511 NHPS range datasets and extracting the amount of mangrove present within each range. The assumption is that the more mangrove forests are present in a defined NHPS range, the higher the chances of the NHPS utilizing the mangrove forest. The NHPS database was subset into endangered status, family, genera, and species, allowing for reporting of each category. The initial processing allows for global-level analysis. The second step is to repeat this process at the country level. This time the unique identifier is NHPS/country instead of only NHPS, and this process allows for reporting the mangrove/NHPS potential interactions at the national level. The penultimate step is to assess the interaction between NHPS and mangrove forests at the local scale and across international or other administrative boundaries. The method selected was to drape a 10 km by 10 km fishnet across the entire universe of NHPS ranges. The fishnet consists of 397,232, 100 km^2^ unique cells. It allows for the depiction of potential NHPS and mangrove forest interaction at this highly granular scale that is not controlled by administrative boundaries. Finally, when localized clusters of interaction were observed, we used global databases, local databases, and a literature review to determine the protected status of these localized potential NHPS/mangrove forest interaction hotspots. Our calculations exclude duplicate counting when two or more NHPS ranges cover the same mangrove stands.

To supplement the geospatial analyses, we conducted a literature search for all the NHPS with mangrove forests in their range, as returned by the geospatial analysis, to assess if the NHPS are demonstrated to use the mangrove forest as habitat. This process is defined in detail above. Habitat is defined as utilizing mangrove forests for foraging, mating, nesting, long-term residence, and the regular use of any resource in the mangrove forest. This literature review accounts for observed NHPS interaction within mangrove forests. In contrast, the presented geospatial analyses measure the potential for interaction or the likelihood of interaction between NHPS and mangrove forests. In addition to conducting a literature review of all NHPS that contained mangrove forests, we randomly selected 30 NHPS that the geospatial analyses depicted as having no mangrove forest in their range and reviewed these species for documented mangrove forest interactions. This additional review allows for estimating a false negative rate when NHPS have no mangrove forests in their range, but an interaction between the two has been observed in the data. Additionally, the literature review allows us to estimate a maximum possible false-positive rate, that is, when there is no evidence of the NHPS interacting with mangrove forests despite our analysis showing overlap occurring.

Finally, we run the Spearman Rho correlation test between the mangrove area size and the number of NHPS overlapping that area.

The supporting information material (S1 Appendix) presented provides a robust process-based supplemental methodology that includes reviewing the hardware, defining the software, outlining the processes, and providing the code.

## Results

**Fig 1:**
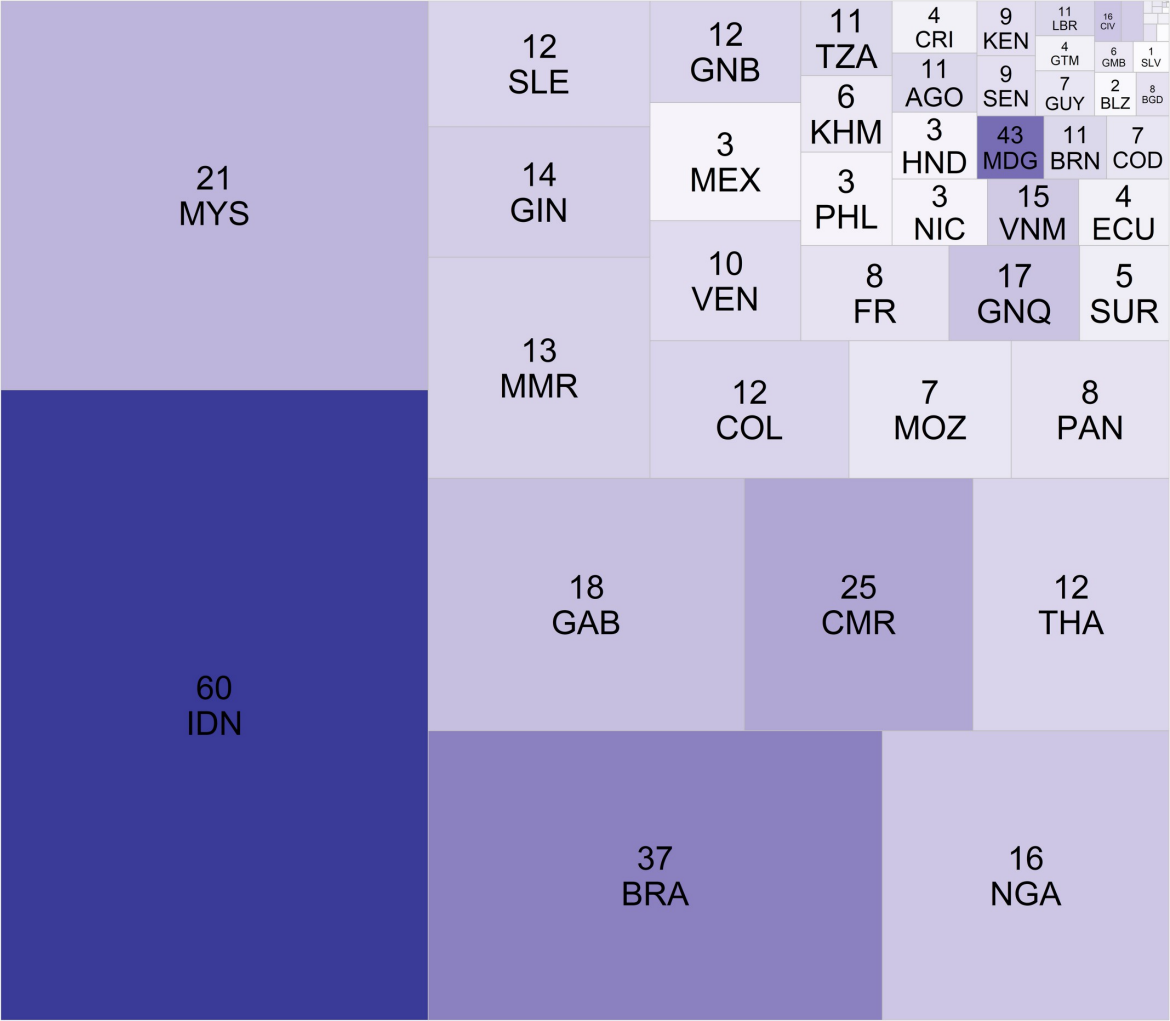
The Tree map represents the mangrove forest area of each country (size of square) and the number of primate species overlapping the mangrove forests in each country (color of square, from light to dark).

As of 2014, the global mangrove forest area within delineated NHPS ranges is 46,426 km^2^. This area constitutes 57 percent of the 2014 total global mangrove forest area. That is, the majority of global mangrove forests are potentially NHPS habitat (Fig 1). Globally, half of all NHPS potentially encounter mangrove forests in their ranges. Of the 511 NHPS with defined ranges, 255, or 50 percent, have delineated ranges that contain at least some mangrove forest (Table 1, S1 Table). The level of mangrove forests in each NHPS range varies substantially. For example, *Trachypithecus delacouri* has only an estimated 108 m^2^, or one pixel, of mangrove forest in its range, whereas *Macaca fascicularis* has 17,233 km^2^ of mangrove forest within its delineated range. Indeed, the range of *M*. *fascicularis* includes 21 percent of all global mangrove forests. When mangrove is present in a NHPS range, the average amount of mangrove forest is 18 km^2^ (n = 255. SD = 9). The Spearman’s rank correlation shows a positive correlation between the mangrove area size and the number of NHPS within these areas, *r*_*s*_ *= 0*.*75*, *p = 0*.*001*, *N = 64* (countries with mangrove and NHPS), at the 0.01 significance level (2-tailed).

**Table 1:** The top 10 of the 255 of the 511 NHPS that overlap mangrove forests, including the mangrove overlap extent, endangered status, species name, and genera information. Continued for all 255 NHPS as S1 Table.

Species	Identifier	Mang. Area	Status	Family	Genus
Macaca fascicularis	12551	17232811994	LC	CERCOPITHECIDAE	Macaca
Macaca nemestrina	12555	12559841649	VU	CERCOPITHECIDAE	Macaca
Trachypithecus cristatus	22035	10967024253	NT	CERCOPITHECIDAE	Trachypithecus
Cephalopachus bancanus	21488	8501179478	VU	TARSIIDAE	Tarsius
Nycticebus menagensis	163013860	7673754445	VU	LORISIDAE	Nycticebus
Nasalis larvatus	14352	7282140979	EN	CERCOPITHECIDAE	Nasalis
Galagoides thomasi	40653	7019822827	LC	GALAGIDAE	Galagoides
Galagoides demidoff	40649	6748763936	LC	GALAGIDAE	Galagoides
Ateles geoffroyi	2279	5416737919	EN	ATELIDAE	Ateles
Cercocebus torquatus	4201	4909461426	EN	CERCOPITHECIDAE	Cercocebus

Continued for all 255 NHPS in S1 Table.

Of the 255 NHPS with delineated ranges that potentially overlap mangrove forests, 101 are part of the Cercopithecidae family, commonly referred to as Old World Monkeys, and 18 are part of the Cebidae family, one of the five families of New World Monkeys (S1 Table). Other significant families present are Atelidae, Cheirogaleidae, Galagidae, Lorisidae, Callitrichidae, Hylobatidae, Tarsiidae, Lemuridae, and Lepilemuridae, with between ten and fifteen species belonging to each of these families present in the mangrove biome (S1 Table). At the genus level, 61 distinct genera contain species that likely overlap mangrove forests (S1 Table). *Macaca* spp., *Presbytis* spp., *Cercopithecus* spp., *Tarsius* spp., *Trachypithecus* spp., and *Lepilemur* spp. are the only genera with ten or more species with ranges overlapping the mangrove forest biome; with 19, 17, 15, 12, 12, and 10 species overlapping, respectively.

**Fig 2:**
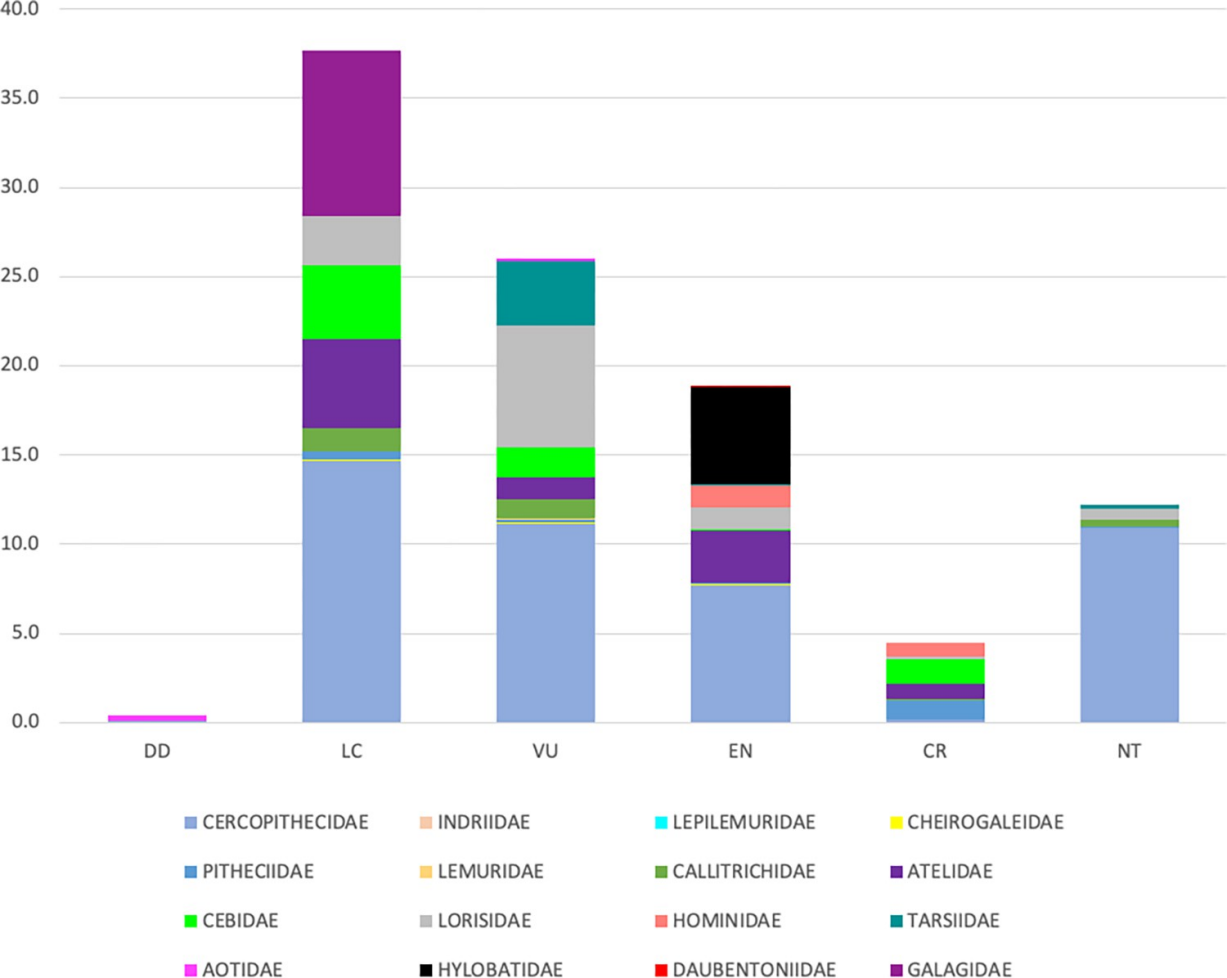
NHPS families within mangroves forests in all countries according to their conservation status. DD–Data Deficient; LC–Least Concerned, NT–Near Threatened, VU–Vulnerable, EN–Endangered, and CR–Critically Endangered.

The total amount of global mangrove forest that overlaps the ranges of NHPS at risk of extinction is 37,798 km^2^ and constitutes 46 percent of the total global mangrove area. This calculation excludes duplicate counting when two or more at-risk NHPS ranges cover the same mangrove stands. Indeed, the majority of NHPS with mangrove forests in their range are at risk of extinction. Of the 255 NHPS whose range contains mangrove forest, 32 of the NHPS are classified as critically endangered and at extreme risk of extinction in the wild, 74 are endangered species and at very high risk of extinction in the wild, and an additional 59 species are listed as vulnerable species and face a high risk of extinction in the wild [14] (S1 Table). That is, 165, or 65 percent, of all NHPS with mangrove forests in their range are at a high risk of extinction in the wild. Additionally, of the world’s 25 most endangered primates at immediate threat of extinction [44], 13 have ranges that include a meaningful amount of mangrove forest. Fig 2 represents the conservation status of NHPS, grouped by family, with ranges that overlap mangrove forests. It is not just NHPS that are at risk of extinction but also the mangrove forests. We find that 69 NHPS ranges overlap mangrove forests threatened with extinction. Indeed, of the 69 NHPS whose ranges overlap mangrove forests threatened with extinction, 52 or 75 percent of the NHPS are themselves at risk of extinction, revealing a potential relationship between mangrove species at risk of extinction and NHPS at risk of extinction.

At the national level, sixty-four countries have NHPS ranges that contain mangrove forests (Table 2, S2 Table). When a country contains NHPS whose ranges overlap mangrove forests, the average number of NHPS present is 9 (n = 557, SD = 10). The five countries with the most overlapping NHPS and mangrove forests are Indonesia, Madagascar, Brazil, Cameroon, and Malaysia, with 60, 43, 37, 25, and 21 NHPS, respectively (Fig 2, Table 2, S2 Table). Indonesia, Brazil, and Malaysia are the top three mangrove holdings nations globally [15]. However, Madagascar is only ranked twenty-first in mangrove holdings and Cameroon eighteenth [15]. The pattern indicates a non-linear relationship between the amount of mangrove present and the number of NHPS potentially present. An extreme example of this relationship is Australia. Australia is the fifth-largest mangrove-holding nation globally [15]; yet, it does not appear in S2 Table, as no NHPS are present in Australia.

**Table 2:** The top 10 of the 64 countries with NHPS that overlap mangrove forests. Complete data for all 64 countries in S2 Table.

Country	NHPS Total
IDN	60
MDG	43
BRA	37
CMR	25
MYS	21
GAB	18
GNQ	17
CIV	16
NGA	16
VNM	15

At the local scale, we use a 10 km by 10 km global grid to determine highly granular locations with relatively high levels of mangrove cover and a relatively high number of NHPS likely present. We find that 10,683 cells contain some mangrove forest and at least one NHPS. The average amount of mangrove in each of these cells is 4.91 km^2^ (n = 10,683, SD = 9.81), constituting slightly less than 5 percent of an entire cell. The maximum amount of mangrove present is 99.92 km^2^, greater than 99.9 percent of an entire cell. The average NHPS number of NHPS present in each of these cells is 4.29 (n = 10,683, SD = 3.24). The maximum NHPS present is 19, with only six of the 10,683 cells having 18 or more NHPS.

**Fig 3:**
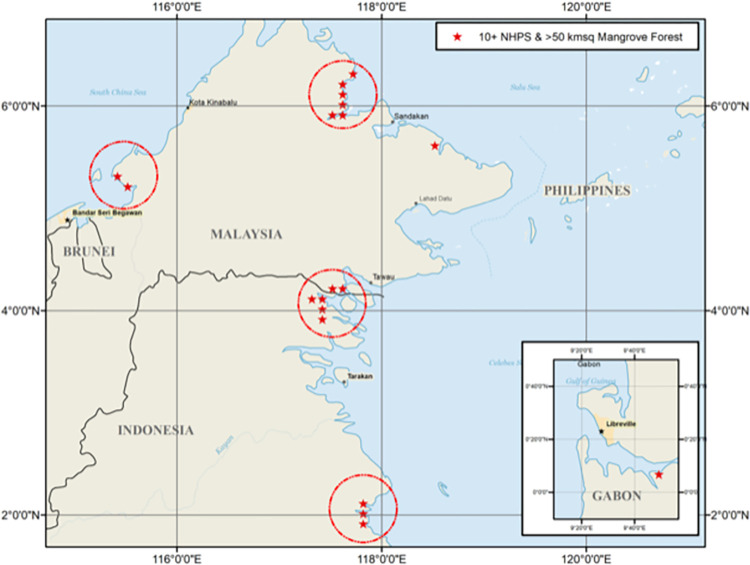
The four distinct clusters and two isolated locations where at least 50 percent of the area is mangrove forest, and more than ten primate species are present.

Within the local analysis, we find four distinct clusters and two isolated locations where at least 50 percent of the local area is mangrove forest, and more than ten primate species are present (Fig 3). All the clusters are in Kalimantan, as it is one of the two isolated locations. The other individual location is in the West African nation of Gabon. The four Kalimantan clusters are located; (i) in Northern Sabah Province, Malaysia, in-and-around the eastern coastline of Beluran District; (ii) at the extreme eastern portion of the border straddling North Kalimantan, Indonesia, and Sabah, Malaysia; (ii) at the mouth of the most northern estuary in the Berau Regency of East Kalimantan, Indonesia; and (iv) within Menumbok Forest Reserve on the Klias Peninsula in Kuala Penyu, Division Pedalaman, Sabah, Malaysia (Fig 3). Prior knowledge of the importance of Kalimantan concerning NHPS use of mangrove forests is known [1], but not at this level of granularity.

Of the identified locations containing a high level of intact mangrove forest and a high NHPS count in Kalimantan and Gabon have mixed protected status. The Malaysian sites are protected as forest reserves [49], a generally lower level of protection within Malaysia designated by the regional forest department rather than the national government. The Kalimantan sites’ protected statuses are undetermined but likely unprotected [48], and the single site in Gabon is protected as it is within Pongara National Park [50].

The literature review of the 255 NHPS with mangrove forests in their delineated range reveals that 147, or 57.6 percent of the NHPS have been directly observed using mangrove forests as habitat (S3 Table). Another 65 of the 255 NHPS, or 25.4 percent, may potentially use mangrove forests as habitat, taking this observed value to 83 percent (S3 Table). Some examples of this wide range between 57.6 percent and 83 percent are species range maps created by a local author overlapping mangrove forests but mangroves not being mentioned [51], terms such as coastal riverine and tropical forests or coastal peat swamps used [52], or even statements that the NHPS has been intentionally relocated outside of its range to a location [53] that is known to contain mangrove forest.

This 83 percent value increases confidence in the validity of the geospatial analyses. Therefore, 17 percent of the NHPS with mangrove forests in their ranges do not have literature supporting their use of the mangrove forest as habitat. Contained within the 17 percent are the false-positive returns from the geospatial analyses. Additional components of this 17 percent are NHPS that have yet to be observed using mangrove forests but likely do and species for which no robust habitat observation yet exists. Finally, none of the thirty randomly selected NHPS returned by the geospatial analyses as not having any mangrove forest in their range have been observed using mangrove forest as habitat according to the literature review. This zero false negative rate indicates that few of the additional NHPS likely use mangrove forests and again increases confidence in the geospatial analysis.

## Discussion

Contrarily to the traditional thought that mangroves are marginal forests, only attracting NHPS when terrestrial habitats are no longer suitable [1, 12, 54, 55], our results support the idea that mangroves are likely a critical NHPS habitat [11, 13, 38]. Although the refuge scenario is likely correct for NHPS in a limited number of individual cases [42], the global patterns of NHPS and mangrove forest interaction show widespread use of mangrove forests as NHPS habitat. Thus, most NHPS appear to utilize mangrove forests as more than a temporary refuge.

Our data revealed an interesting relationship between at-risk mangrove forests and at-risk NHPS. When mangrove forests are endangered, the percentage of at-risk primates with their range in the endangered mangrove forests is 75 percent of all NHPS present. When the mangrove is not at risk of extinction, this figure drops to 50 percent. Such a pattern indicates that NHPS is more threatened when their mangrove habitats are more threatened or even vice-versa. A simple potential cause may be that the driving mechanism of the potential NHPS and mangrove extinction, such as human encroachment, impacts both groups in a similar spatiotemporal manner. An alternate hypothesis is that mangrove forest loss drives NHPS vulnerability in these regions regardless of the mechanism of mangrove forest loss. Indeed, although less likely, it is even possible that NHPS vulnerability may cause mangrove vulnerability via seed dispersion [56] or other unknown means. For instance, in Myanmar, the endangered capped langur population decreased [57] due to a substantial portion of the mangrove forest where they lived being converted into shrimp and salt farming [58]. Similarly, in the Cat Ba langurs in Cua Dong, Vietnam, individual langurs were isolated from the mainland forest due to mangrove destruction [59].

Species range maps with rigid boundaries at discrete locations imperfectly represent the complexity of a range and should not be considered perfect delineations. Both errors of omission and commission likely occur in species range data. Indeed, it has been noted that the single most significant cause of variation amongst species distribution polygons for a single species is the model utilized [60], as opposed to the input data utilized. Additionally, in this use case, care should be taken when assuming that small range overlaps, such as a few hundred meters, are actual use of mangrove forests by NHPS as small overlaps could result from spatial error and not actual interactions. The literature review table can help determine if the reported small overlaps represent mangrove forests used by NHPS. Cross-validation with non-IUCN data, such as the recently released expert mammalian range maps [61], is an obvious step in expanding this methodology and increasing knowledge of the use of mangrove forests by NHPS.

This analysis makes it possible to establish priority global, regional, and local areas for field validation and more direct conservation strategies for primate species that are endangered, critically endangered, and at extinction risk in mangroves. Systematically mapping mangroves in primate home ranges would provide better validation to our knowledge of their presence in mangrove forests. Likewise, surveying primates while mapping mangroves can improve global conservation actions due to the importance of the role of wildlife in habitat restauration [62]. Mangrove forests appear to be a critical habitat to more generalist primates, mainly if we assume that terrestrial forests adjacent to mangroves are degraded and diminished in many areas, exposing the importance of mangroves as suitable habitats for primates. The significant overlap of mangroves with primate species enhances the necessity to combine conservation efforts to protect both.

Finally, this research presents a conservation-connection approach between animal species and forests that might potentially help each other on conservation plans. While NHPS are critical for forest regeneration [63], mangrove forests are critical as habitat, refugee, and food security [29]. Additionally, this connection presents an interesting insight into how preserving NHPS may additionally resist climate change. Mangroves contain some of the highest carbon stocks per hectare of any forest type globally [64], storing between 4.19 Pg and 5.02 Pg of carbon [65, 66]. Protection of the mangrove forest biome, driven by concern for NHPS habitat conservation, could not only mitigate global changes but preserve the habitat of half of the closest living non-human relatives.

## Supporting information

S1 Table The 255 of the 511 NHPS that overlap mangrove forests, including the mangrove overlap extent, endangered status, species name, and genera information. (CSV)

S2 Table The 64 countries with NHPS that overlap mangrove forests. (CSV)

S3 Table The literature review of the 255 NHPS with mangrove forests in their range. (CSV)

S1 Appendix (PDF)
