# Supplementary material for: Establishing the relationship between non-human primates and mangrove forests at the global, national, and local scales
Source: PLoS One. 2022 Nov 11;17(11):e0277440. doi: 10.1371/journal.pone.0277440 (PMC9651587; doi:10.1371/journal.pone.0277440)
Supplement: S1 Appendix — (PDF) [file pone.0277440.s004.pdf]

***S1 Appendix***

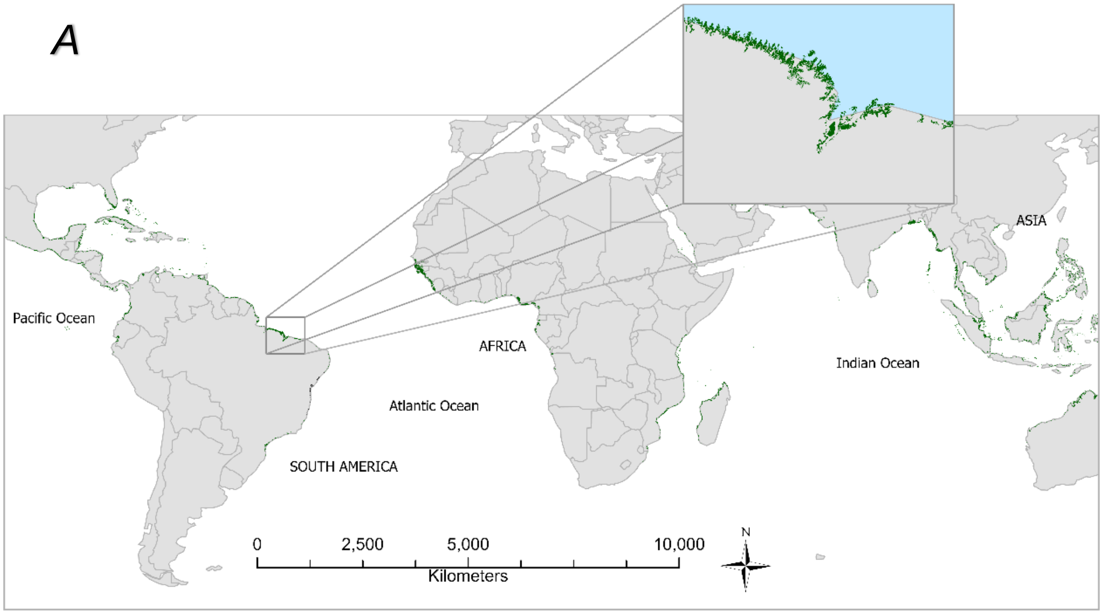


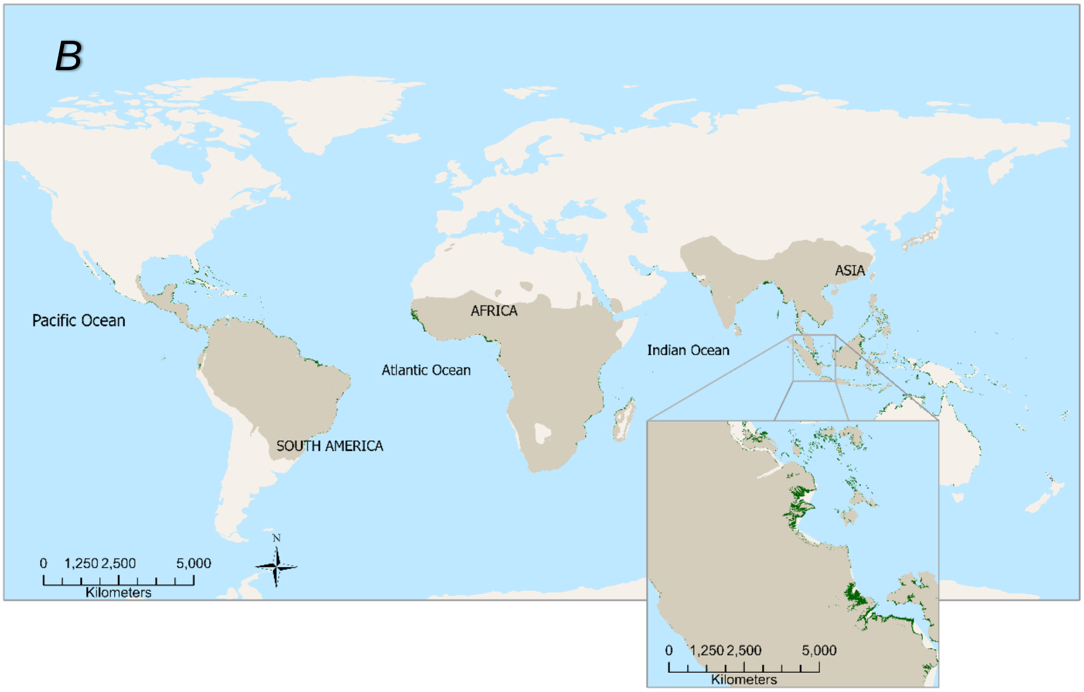


Figs S1 A) Mangrove global data, and S1 B) Mangrove global data overlaying the primate IUCN global data

***Supplemental Methodology***

The CGMFC-21 2014 mangrove dataset is stored in a singular 617 GB raster file and contains over 330 billion pixels (1). Of the 330 billion pixels, approximately 121 million pixels contain data on the number of square meters of mangrove forest. The 511 IUCN NHPS ranges consist of 511 separate and overlapping multipart polygons constructed from over 1.6 million coordinate pairs (1).

The primary analytical task is to determine the amount of mangrove area within each IUCN NHPS polygon, a relatively straightforward task. However, the volume of data is large enough that traditional GIS methods are insufficient as they require many months of processing time per iteration. Therefore, an alternate solution relying on data restructuring and parallel processing is necessary.

Preprocessing included polygon verification and repair, which accounted for correcting the polygons for invalid features, data reprojection, and subsetting the NHPS range data. Once preprocessing was complete, the first step is to convert the CGMFC-21 raster mangrove areas to 121 million coordinate pairs, representing the center point of each pixel (SQL CODE STRUCTURE ONE). The mangrove raster file has intermittent values, as mangroves only exist in discrete locations. Therefore approximately 99% of the CGMFC-21 points have no mangrove area associated with them. An indexing scheme stores the coordinate pairs within tiles, most of which are null.

Once the 121 million coordinate pairs of mangrove forest areas are created, the next step overlays the IUCN NHPS ranges and the mangrove forest data. To better facilitate the overlay, the 511 polygons were subdivided into 195,000 smaller polygons so that the overlay function would evaluate many smaller and simpler polygons (SQL CODE STRUCTURE TWO). With smaller and simpler polygons, the overlay operation can take advantage of a spatial index. The large and complex IUCN NHPS ranges are simplified into dozens of smaller areas, forcing the containment query to process efficiently.

Once the data are overlaid, the script calculates the number of mangrove points in each polygon, the sum of the mangrove forest areas in each polygon, and the average size of each polygon mangrove pixel (SQL CODE STRUCTURE THREE). This code is implemented in parallel across all available processors. With overlapping IUCN ranges, large numbers of vertices, and many polygons covering many tens of thousands of square kilometers, a traditional GIS approach is unsuitable. The large expanse of the polygons has too many contained points to calculate a containment operation efficiently.

Finally, hundreds of thousands of polygons are grouped back into their 511 unique species polygons (SQL CODE STRUCTURE FOUR).

The above process produces results on a global scale for all NHPS. This process can result in duplicate counting of mangrove forest areas when NHPS ranges overlap. Such counting is desirable in most cases but undesirable when examining NHPS at the family level. Therefore, the process is repeated on a single dissolved NHPS polygon that facilitates the non-duplicate counting of mangrove forest areas (1). For endangered primate species, the 25-most endangered NHPS, and other NHPS subsets such as genera, the outlined process is merely repeated on subsets of the NHPS data (1). At the national level, the NHPS species data was intersected with all current nation-state boundaries to create a mapping unit consisting of unique country/NHPS polygons instead of merely NHPS polygons as used in the global analysis (1). Finally, the 10 km fishnet was intersected with the NHPS polygons to account for potential local

NHPS and mangrove forest interactions (1). The national and local functions require reaggregation of NHPS data that may be artificially split due to introducing an additional set of boundaries.

Preprocessing of data, mapping of the results, and statistical operations were conducted in QGIS (2), ESRI ArcPro (3), and GDAL (4). Processing of the data used Manifold GIS Version 9 (5), which provides an SQL engine for processing very large data stores and has built-in parallel processing capabilities.

The full data and replication repository, including all code, input geospatial data, and output tables, can be found at https://doi.org/10.7910/DVN/QRYBYR (1).

SQL CODE STRUCTURE ONE (1)

Tiles containing raster values in the CGMFC-21 data store were identified using the *TileToValues* function, scanned for individual pixels, and converted to points using the *GeomMakePoint* function. The code permits the tile structure to filter out only those sections that contained pixels with values greater than zero and reduces the volume of data by greater than 90 percent.

```
SELECT GeomMakePoint(VectorMakeX2(tx*128+x, ty*128+y)) AS [geom],
Value
INTO [pixelpoints]
FROM (
       SELECT x AS tx, y AS ty, SPLIT CALL TileToValues(tile)
       FROM [a2014CGMFC21]
       WHERE TileValueMin(tile) > 0 AND TileValueMax(tile) < 901
       )
WHERE value > 0;
```

SQL CODE STRUCTURE TWO (1)

The IUCN polygons are split into hundreds of thousands of smaller polygons *GeomToBranches* function.

```
SELECT [IUCN_Primates.Shape] .*, SPLIT CALL
GeomToBranches(GeomToConvex([Shape], 0)) AS shape

INTO subdividedgeom

FROM [IUCN_Primates]
```

SQL CODE STRUCTURE THREE (1)

The SQL function *GeomOverlayContainedPar* performs the point-in-polygon function. The point-in-polygon *GeomOverlayContainedPar* function was embedded into a standard *Update* function to update the subdivided polygons. Restructuring the data into tiles and utilizing parallel processing reduced the process from days to under 24 minutes.

```
UPDATE (
 SELECT
  t.[mfd_id] AS tkey0,
  t.[numpoints] AS t0, t.[averagehectares] AS t1,
       t.[sumhectares] AS t2,
  s.s0, s.s1, s.s2
 FROM [subdividedgeom] AS t LEFT JOIN (
  SELECT
   [s_mfd_id] AS skey0,
   Count([o_mfd_id]) AS s0,
   Avg([o_Value]) AS s1,
   Sum([o_Value]) AS s2
  FROM CALL GeomOverlayContainedPar(
[subdividedgeom],                     [landpixels], 0,
ThreadConfig(SystemCpuCount())
                                        )
  GROUP BY [s_mfd_id]
 ) AS s ON t.[mfd_id] = s.skey0
) SET
 t0 = s0, t1 = s1, t2 = s2;
```

SQL CODE STRUCTURE FOUR (1)

As all the subdivided polygons contain duplicate ids from the parent IUCN ranges from which they were derived, the results needed to be summarized by the original IUCN id, using a simple *GROUP BY* function.

```
SELECT id_no,sum([sumhectares]) AS sumhectares,
  avg([averagehectares]) AS averagehectares,
  sum(numpoints) AS totalpixels
INTO monkeytable
FROM [subdivided]
GROUP BY id_no
```

## References


1. S. Hamilton, Replication Data for: MangPrim-21: Establishing the Relationship between Non-Human Primates and Mangroves Forests at the Global, National, and Local Scales. Harvard Dataverse. https://doi.org/10.7910/DVN/QRYBYR.
2. QGIS Development Team (2017) QGIS. (Open Source Geospatial Foundation, Dover, DE).
3. ESRI (2021) ArcPro 2.7. (ESRI, Redlands, CA).
4. GDAL Development Team (2016) Geospatial Data Abstraction Library. (Open Source Geospatial Foundation, Dover, DE).
5. Manifold Software Limited (2021) Manifold 9. (Manifold Software Limited,, New York, NY).
